# Differences in Self-Concept and Its Dimensions in Students of the Third Cycle of Primary School, Obligatory Secondary Education, and Baccalaureate

**DOI:** 10.3390/healthcare11070987

**Published:** 2023-03-30

**Authors:** Angel Denche-Zamorano, Noelia Mayordomo-Pinilla, Carmen Galán-Arroyo, Carlos Mañanas-Iglesias, Jose C. Adsuar, Jorge Rojo-Ramos

**Affiliations:** 1 Faculty of Education, Psychology and Sport Sciences, Universidad de Huelva, 21007 Huelva, Spain; 2 Promoting a Healthy Society Research Group (PheSO), Faculty of Sport Sciences, University of Extremadura, 10003 Cáceres, Spain; 3 Physical and Health Literacy and Health-Related Quality of Life (PHYQoL), Faculty of Sport Science, University of Extremadura, 10003 Cáceres, Spain; 4 Social Impact and Innovation in Health (InHEALTH) Research Group, Faculty of Sport Sciences, University of Extremadura, 10003 Cáceres, Spain; 5 Physical Activity for Education, Performance and Health, Faculty of Sport Sciences, University of Extremadura, 10003 Cáceres, Spain

**Keywords:** self-concept, educational stage, physical self-concept, AF-5 scale, students

## Abstract

Self-concept can be defined as a structured, multidimensional, and evolving construct that constitutes all the beliefs that an individual has about him/herself. Among its dimensions is the physical dimension that encompasses perceptions of physical attractiveness, self-esteem, and physical condition. The purpose of this cross-sectional study was to look for differences between the educational stages from the third cycle of primary school and high school, as well as to study the possible correlations between the age groups and the dimensions of the scale. The AF-5 scale was used to measure self-concept, the Kolmogoronov–Smirnov test was applied to determine the normality of the data, Kruskall–Wallis to identify the differences between the dimensions of the scale and the educational stages, and Spearman’s Rho for correlations between dimensions and age groups. Significant differences were found in the academic, emotional, family, and physical dimensions between educational stages and between the scale as a single construct. Significant inverse correlations were also found between age groups and dimensions. Overall self-concept decreases with age and varies according to the educational stage.

## 1. Introduction

Self-concept is a term that has been gaining importance in recent years in the field of psychological research (multidisciplinary, experimental, developmental, educational, or social), together with education educational research, family studies, among other areas, being separately studied or in relation to multiple variables and themes [1,2,3,4,5]. It can be defined as the set of beliefs that an individual has about him/herself at a given moment, conditioned by the positive or negative feelings he/she has about him/herself, which also builds the personality and conditions the social and emotional development of the subject [6,7]. These beliefs are built over time, determined by the lived experiences of each individual, influenced by the social and cultural environment in which he/she finds him/herself [8,9].

This construct has been studied by multiple researchers, who have identified different components that make it a multidimensional element, which constitutes the set of thoughts that a person has about him/herself, erecting his/her personality [7], stating that it is a structural, multidimensional, hierarchical, and evolutionary model [10]. Self-concept is composed of five dimensions: social, emotional, academic/work, family, and physical [11]. The first dimension of this construct refers to the individual’s perception of the quality of his or her actions in his or her own environment; the social dimension refers to how the individual perceives his or her interaction in the immediate social environment and in interpersonal relationships; the emotional dimension encompasses the individual’s view of his or her emotional responses to specific situations that involve a commitment; the family dimension refers to the perception of the degree of involvement in his or her family environment; finally, the physical dimension encompasses the individual’s opinion of his or her physique and physical condition [12]. This last dimension, physical self-concept, is of utmost importance in the adolescent stage, because it is the period when personality is formed and is closely linked to mental health and psychological well-being [13,14], and in addition, the scientific community has observed that relationships are established between physical appearance, physical self-concept, self-esteem, and behavior [14,15,16], therefore, if a correct self-concept is developed, positive functioning in all social and personal dimensions can be achieved [17].

Physical self-concept, therefore, is an important factor in the mental health of adolescents. The authors define this dimension as the person’s view of his or her physical appearance and physical capacities, conditions, and abilities [15,18]. The scientific literature identifies relationships between this construct and physical practice [9,19] because physical activity increases physical self-concept, at least in the short term [20], although they also identify relationships between this construct and performance in PE, sports practice, eating disorders, and other psychological disorders [21,22]. In this sense, physical education (PE) is an effective tool for providing adolescents with resources to improve their physical self-concept and avoid these disorders, because PE is an ideal medium for promoting healthy lifestyles and positive habits [23]; other authors state that self-concept conditions behavior, therefore, adolescents with positive self-concepts will be more self-confident and will act with more determination [24]. Likewise, adolescence is the period of time in which habits for the future are generally adopted, so it would be easier for them to develop positive habits that are maintained over time, among which are physical activity and physical self-concept [8,25].

On the other hand, being an evolutionary construct, the research community has sought relationships between the five dimensions of self-concept and age, inquiring into the possible changes related to the passage of time or the educational stage. Changes in self-concept according to age are known and there are important cognitive and life changes, such as metacognition or social changes among the subjects attending classes in the different stages. In this sense, some studies have not been able to confirm, or only partially confirmed, the hypothesis that self-concept is related to age, because not all dimensions of self-concept have the same behavior [8,26]. Therefore, although self-concept may be age-related and may change over the years, this relationship could be conditioned by the evolution of only some of its dimensions, by different evolutions of these dimensions over time, or by evolutions of these dimensions according to changes between educational stages.

In Spain, the educational system is structured as follows: primary education, which is compulsory and free of charge, contains three cycles of two courses each for students aged 6 to 12 years (first cycle, students aged 6 to 8 years; second cycle, students aged 8 to 10 years; third cycle, students aged 10 to 12 years); compulsory secondary education (ESO), consisting of two cycles of two compulsory courses for students aged 12 to 16 years (first cycle, 12 to 14 years; second cycle, 14 to 16 years). Finally, the baccalaureate is voluntary, consisting of two academic years for students between 16 and 18 years of age [27]. In these educational stages, PE is a compulsory subject in all of them, except in the second year of the baccalaureate.

To our knowledge, no article has investigated the evolution of self-concept and its dimensions in the different educational stages, analyzing the possible differences between stages. For this reason, the main aims of this study were to investigate the possible associations between the different dimensions of self-concept and the educational stages of the third cycle of primary school, ESO, and baccalaureate, as well as to analyze the relationships between the age of the students and these dimensions of self-concept.

## 2. Materials and Methods

### 2.1. Design

This research was developed following a cross-sectional study design based on data obtained from questionnaires administered to children and adolescents (between 10 and 18 years old), students from public schools of primary education, secondary education and/or baccalaureate in Extremadura (Spain).

### 2.2. Participants

The total sample consisted of 960 students from different schools in Extremadura (Spain), in both rural and urban centers. Rural centers were defined as those located in municipalities with less than 20,000 inhabitants and urban centers as those located in towns with more than 20,000 inhabitants according to the Cáceres (Extremadura, Spain) Provincial Council (Diputación de Cáceres: https://www.dip-caceres.es/, accessed on 7 January 2023). The sample size was determined according to the non-probabilistic sampling method based on convenience sampling [28].

To participate in this research, the students had to meet a series of inclusion criteria: (1) Informed parental consent. (2) Attending physical education in a public school in Extremadura in the third cycle of primary education, ESO, or baccalaureate (from 10 to 18 years of age). Therefore, students from the first and second cycle of primary education (students aged between 6 and 9 years old) in public schools in Extremadura were not invited to participate, being excluded in a phase prior to the administration of the questionnaires. Similarly, students in higher grades, who did not have physical education as part of their curriculum—and therefore did not take the subject—were not invited to participate in the study and were also excluded before the questionnaires were administered.

Regarding ethical provisions, this research was conducted in accordance with the Declaration of Helsinki and the protocol was approved by the Bioethics Committee of the University of Extremadura (Registration Code 71/2022).

### 2.3. Procedure

To obtain the sample, access was gained to the database of the Department of Education and Employment of the Regional Government of Extremadura, which contains data on the public schools in which primary education (from 6 to 12 years of age), secondary education (from 12 to 16 years of age), and baccalaureates (from 16 to 18 years of age) are taught.

To contact the selected schools, an e-mail was sent to the physical education teachers at these schools containing the purposes of the research, parental informed consent, and a copy of the model instrument that would later be applied. The teachers were also asked to send an e-mail if they were willing to collaborate in order to arrange an appointment for a person responsible for the research to come to the center to apply the questionnaire to those students who had the informed consent of the parents. At the time of administering the questionnaire, after collecting the signed informed consents, each student was provided with an electronic device so that they could access the questionnaire through a link, elaborated on the Google Forms platform, and then explain each element of the form with the aim of eliminating all possible doubts regarding what was requested in each item. An electronic questionnaire was chosen in order to facilitate the storage and subsequent handling of the responses obtained to ensure the process and save paper, time, and costs. Once the data had been collected from all the participants, after the questionnaires had been administered in all the schools that agreed to participate in the study, data curation, as well as data processing and anonymization were carried out by the researchers C.M-I. and C.G-A. The subsequent data analysis was carried out, blinded, by J.R-R.

### 2.4. Instruments

A questionnaire with four questions was created to extract information on the sociodemographic data of the participants: sex (male or female), age (years), educational level (third cycle of primary education, first cycle of compulsory secondary education, second cycle of compulsory secondary education, or baccalaureate), and location of the center (if the school was situated in a municipality with more than 20,000 inhabitants, it was considered an urban school. If the school was situated in a municipality with less than 20,000 inhabitants, it was considered to be a rural school).

On the other hand, a multidimensional theoretical model of self-concept was applied, known as the AF-5 self-concept scale [11], this model is designed to assess self-concept in five dimensions: (1) academic-work (items: 1, 6, 11, 16, 21, and 26), (2) social (items: 2, 7, 12, 17, 22, and 27), (3) emotional (items: 3, 8, 13, 18, 23, and 28), (4) family (items: 4, 9, 14, 19, 24, and 29), and (5) physical (items: 5, 10, 15, 20, 25, and 30); where each of the dimensions has 6 items for a total of 30 items [29]. In addition, the total score of the questionnaire, which is the sum of all items and assesses the overall self-concept is also obtained. To obtain the scores for each item, the Likert scale is used, with values from 1 to 5, where 1 is “totally disagree” and 4 is “totally agree”. Therefore, 1 is the best self-concept, and 5 is the best. Regarding the psychometric properties of the instrument, values above 0.71 are reported by the authors in all dimensions; additionally, the items of the scale as a whole scored 0.78, expressing that all items assess self-concept [7,11].

### 2.5. Statistical Analysis

The Statistical Package for Social Sciences (IBM SPSS Statistics, version 26, Chicago, IL, USA) was used for data analysis [30]. With the aim of finding out if the data followed a normality assumption, the Kolmogoronov–Smirnov test was applied, reporting that the data did not comply with normality (*p* = 0.000), determining that non-parametric tests would be applied; on the other hand, Cronbach’s alpha was used with the purpose of quantifying the reliability of the dimensions of the scale applied in this research.

Subsequently, the Kruskall–Wallis test was applied to identify possible differences between the dimensions of the scale and the educational stages. The post-hoc test was also implemented in order to compare the educational stages and identify significant differences. Finally, Spearman’s Rho test was used to test the associations between the dimensions and the ages of the participants.

In all statistical analyses for this study, a *p*-value lower than 0.05 was considered statistically significant.

## 3. Results

### 3.1. Sample Characterization

The total sample, consisting of 960 students between the ages of 10 and 18, was distributed as follows: 48.2% were girls and 51.8% boys, constituting a fairly balanced sample with respect to the number of individuals per sex. Regarding the educational stage, 15.2% (*n* = 146) belonged to the third cycle of primary education, 27% (*n* = 259) to the first cycle of ESO, 28.9% (*n* = 277) to the second cycle of ESO, and finally, 29% (*n* = 278) to baccalaureate. As for the location of the center, 22.9% (*n* = 220) studied in a rural center, while 77.1% (*n* = 740) did so in an urban center.

The average ages of the participants according to educational level were 10.74 years old (sd = 0.70), for the third cycle of primary education; 13.60 years old (sd = 0.78) for the first cycle of compulsory secondary education 15.52 years old (sd = 0.64) for the second cycle of compulsory education and 16.54 (sd = 0.67) years old for baccalaureate.

The sociodemographic data of the sample characterization are shown in Table 1.

### 3.2. The AF-5 Questionnaire

Table 2 shows the results obtained from the AF-5 scale, with the differences between the dimensions (academic, social, emotional, familiar, and physical) according to educational stage. Data are shown as median and standard deviation. Kruskall–Wallis was applied to identify significant differences.

In dimension 1, “Academic”, statistically significant differences were found in the scores obtained by the group of students in the third cycle of primary education with respect to the groups in the first cycle of compulsory secondary education (*p* < 0.001), the second cycle of compulsory secondary education (*p* < 0.001) and baccalaureate (*p* < 0.001). Students belonging to the third cycle of primary education obtained the highest scores in this dimension (Table 2).

In dimension 3, “Emotional”, statistically significant differences were found between the scores obtained by the students in the first cycle of secondary education and the students who studied baccalaureate (*p* = 0.022). Students who attended high school scored higher in this dimension (Table 2).

With respect to dimension 4, “Family”, statistically significant differences were found between the scores obtained by students in the third cycle of primary education and students belonging to the first cycle of compulsory secondary education (*p* < 0.001), the second cycle of compulsory secondary education (*p* < 0.001), and students studying baccalaureate (*p* < 0.001). Students in the third cycle of primary education scored higher in this dimension (Table 2).

In dimension 5, “Physical”, statistically significant differences were found between students in the third cycle of primary education and students in the second cycle of compulsory secondary education (*p* = 0.017). Once again, students in the third cycle of primary education obtained the highest scores (Table 2).

Finally, if we take the five dimensions as a single construct (self-concept), the following statistically significant differences were found between the scores obtained by the following groups: students who studied the third cycle of primary education and students in the first cycle of Compulsory Secondary Education (*p* < 0.001), students in the third cycle of primary education and those who studied the second cycle of compulsory secondary education (*p* < 0.001), and students in the third cycle of primary education and high school students (*p* < 0.001). Finally, statistically significant differences were also found between the scores obtained by students in the second cycle of compulsory secondary education and students in high school (*p* = 0.017). It was the students in the third cycle of primary education who obtained the highest score in the self-concept construct (Table 2).

Table 3 shows the correlations between self-concept and its dimensions according to the AF-5 self-concept scale and the age groups of the students, obtained after the application of Spearman’s Rho test.

Significant correlations are found in several dimensions. In dimension 1, “Academic”, there is a significant inverse correlation (rho: −0.19, *p* < 0.001), as well as in dimension 4, “Family” (rho: −0.19, *p* < 0.001). This means that as age increases, the scores obtained in these dimensions of the questionnaire will be lower, indicating that the older the student is, the worse his conception of his academic and family self-perception is. On the other hand, if the questionnaire is observed as a whole and considered as a construct, significant inverse correlations also appear (rho: −0.12, *p* < 0.001), which means that the older the student is, the worse general self-perception he/she has about him/herself. However, no statistically significant relationships were found between age and the “Social”, “Emotional”, and “Physical” dimensions (Table 3).

## 4. Discussion

### 4.1. Main Findings

The main objective of this research was to analyze the possible relationships between the dimensions of self-perception according to the AF-5 scale scores, specifically to analyze the differences in the “physical” dimension and the different educational stages between the third cycle of primary education and high school, as well as the possible correlation between the five dimensions and the age groups. The literature is limited in this aspect because there are not many articles on the differences between the dimensions according to educational stage, but they are more focused on the correlations and differences between genders within the same age group.

Among the main results of the AF-5 scale, significant differences appear in dimension 1, “academic”. The importance of this dimension lies in the fact that it is essential to understand the students’ behavior with respect to their opinion of themselves [31]. Students in the third cycle of primary education scored higher than students in both cycles of ESO and those in baccalaureate, indicating that they have a better self-perception about academics than the other groups. In line with these results, researchers have reported a negative trend in the self-perception of this dimension in the transition from primary to secondary education [32,33,34].

In dimension number 2, “social”, no significant differences are found in the different educational stages, although in general terms, they have a fairly high score. In this line, studies report that this dimension, together with the “family” dimension, are the ones with the highest scores, establishing that students do not have too many difficulties establishing interpersonal relationships and maintaining them over time, because they rely on their peers [35,36,37].

Next, in dimension 3, “emotional” encompasses the student’s own perception of the emotional responses he/she gives in day-to-day situations [31]. In the results of this dimension, the lowest scores of all dimensions are obtained in all educational stages, in line with what has been found in other publications [38,39] On the contrary, other studies show that in general, students have a good perception of the emotional dimension [37]. Significant differences also appear between the group of the first cycle of ESO and baccalaureate, with students in the latter grade scoring higher. In line with these results, those obtained by Lope Álvarez et al. report a higher score in baccalaureate students than in ESO students [40] in the emotional dimension of the scale.

Regarding the fourth dimension of the scale, “family”, significant differences are reported among students of all educational stages, with students in the third cycle of primary school scoring higher. It is the dimension with the highest score in general, as reported by other studies [37,38,41]. These high scores in the third cycle of primary school in relation to the rest of the educational stages may be due to the detachment from parents that occurs in adolescence, increasing social contact, and decreasing family contact [35,42,43].

Finally, in the “physical” dimension, significant differences appear between students in the third cycle of primary school and those in the second cycle of ESO, with the former having a higher score. This can be explained by the fact that the behavior of this dimension fluctuates over time, so that from adolescence onwards the score falls, although it is consolidated again in adulthood [8,41]. The literature consulted shows that there is a high degree of association between this dimension and the subject of PE [44], reporting that those who perform more physical activity are those who obtain higher scores in this dimension, that is, they have a greater self-concept of this section, acting with greater confidence in their abilities and in themselves, in addition to a greater degree of autonomy, self-esteem, and a better perception of their physique [17,21,45,46]. In this line, several researchers have implemented different physical activity programs in physical education classes and obtained improvements in physical self-concept in all its subdimensions [47,48,49].

Overall, significant differences are reported between educational stages with respect to the AF-5 form as a whole, because self-concept, as a single construct, varies over time as do its dimensions, especially in adolescence, which is when it suffers the most [43,50].

Regarding age groups and dimensions, significant inverse correlations were found in dimensions 1, “academic”, and 4, “family”. These correlations reveal that the older the students are, the lower their scores in these dimensions, and the worse their self-concept. In line with these revelations, several studies obtained similar results [35], explaining that the “academic” dimension is related to the “family” and “emotional” dimensions so that during adolescence both suffer, affecting the academic dimension [35]. 

If we analyze the results of the AF-5 scale as a single construct, significant inverse correlations are also found, establishing that self-concept in general decreases as the age of the person increases, in this case, in students. With the same characteristics, studies report a marked decrease in self-concept during the first cycle of ESO, maintained during the rest of adolescence [51]. On the other hand, other studies report that the age variable lacks explanatory capacity regarding the behavior of self-concept [50,52].

Considering that self-concept seems to deteriorate as students advance through the educational stages and increase in age—and that deterioration of this seems to have a negative influence on health, physical activity, or academic performance, among other aspects—it seems important that it should be taken into account by teachers and other professionals involved in the education of young people and adolescents.

### 4.2. Limitations and Future Lines

As a limitation, this study was carried out with students exclusively from the region of Extremadura, so the results may be influenced by sociocultural factors. On the contrary, this provides an opportunity for further research in this line of work and offers us the possibility of future lines of research. Also, the data were electronically collected, with the disadvantages that this entails. It might be interesting to assess whether the results would have been the same if the questionnaires had been administered in a different format, or even if interviews could have been conducted, or if a qualitative study design had been used. In the future, it would be interesting to increase the socio-demographic data of the participants in order to identify other variables that could explain self-concept behaviors, as well as to extend the sample to other communities, at a national level. Moreover, as mentioned in the theoretical background, there are a large number of variables that can affect people’s self-concept. Among them are academic performance, sports performance, physical activity level or sports practice, possible special needs, or some kind of physical or mental disorder. Unfortunately, these were not considered for this study and present an important limitation, as they may be contaminating variables. At the same time, it could be a very interesting object of study for future research, developing these aspects. Due to the actual design of the research, in accordance with one of the main limitations of cross-sectional studies, it is not possible to establish cause–effect relationships, which is why this study is only descriptive and not explanatory. As a line for the future, it would be advisable to carry out research based on other types of design that would allow causal relationships to be established. In addition, the multidimensional model of self-concept used in our research, based on the results obtained in the AF-5 self-concept scale, is not the only model that researchers have used to assess self-concept, and there are other different models, assessed by other questionnaires. Using other questionnaires, or models to determine the self-concept of children and adolescents, we could have found different results. In future research, it would be useful to explore these other existing possibilities for assessing self-concept, beyond the one used in our study.

## 5. Conclusions

As for the general conclusions, self-concept as a single construct is different according to the educational stage in which they are, having a higher score in students in the third cycle of primary education, decreasing until the second cycle of ESO and stabilizing in baccalaureate. On the other hand, significant differences were found in the physical self-concept dimension between students in the third cycle of primary education and those in the second cycle of ESO. In addition, significant inverse correlations were found between the AF-5 scale and the age groups, decreasing as age increases.

## Figures and Tables

**Table 1:** Sample characterization (N = 960).

Variable	Categories	*n*	%
Sex	Men	497	51.8
	Women	463	48.2
Educational stage	Third cycle of primary education	146	15.2
	First-cycle compulsory secondary education	259	27
	Second-cycle compulsory secondary	277	28.9
	Baccalaureate	278	29
Province of the school	Rural	220	22.9
	Urban	740	77.1
Variable	Categories	M	SD
Age	Third cycle of primary education	10.74	0.70
	First cycle of compulsory secondary education	13.60	0.78
	Second cycle of compulsory secondary	15.52	0.64
	Baccalaureate	16.54	0.67

N: number; %: percentage; M: mean; SD: standard deviation.

**Table 2:** AF-5 descriptive analysis and differences of each Dimension.

	Total	Education Stage				
Dimensions	M (SD)	Third Cycle of Primary Education	First-Cycle Compulsory Secondary Education	Second-Cycle Compulsory Secondary	Baccalaureate	*p*
1. Academic	3.72 (0.78)	4.18 (0.63)	3.65 (0.77)	3.57 (0.74)	3.69 (0.80)	<0.001 *
2. Social	3.78 (0.70)	3.75 (0.64)	3.83 (0.68)	3.69 (0.73)	3.83 (0.73)	0.032
3. Emotional	2.73 (0.79)	2.70 (0.72)	2.63 (0.79)	2.75 (0.76)	2.84 (0.84)	0.030
4. Familiar	4.38 (0.73)	4.72 (0.37)	4.42 (0.74)	4.29 (0.76)	4.26 (0.78)	<0.001
5. Physical	3.50 (0.77)	3.61 (0.69)	3.52 (0.80)	3.38 (0.75)	3.53 (0.78)	0.012
AF-5	3.62 (0.40)	3.79 (0.34)	3.61 (0.40)	3.54 (0.38)	3.63 (0.41)	<0.001

*p* is significant at the * *p* < 0.016. Me = median value; SD = typical deviation. Each score obtained is based on a Likert scale (1–5): 1 being “Strongly Disagree”, 2 “Partially Disagree”, 3 “Neutral”, 4 “Partially Agree”, and 5 “Strongly Agree”.

**Table 3:** Correlations between the dimensions and the age group variable.

Dimensions	Age *ρ* (*p*)
(1) Academic	−0.19 (<0.001)
(2) Social	0.04 (0.162)
(3) Emotional	0.03 (0.235)
(4) Family	−0.19 (<0.001)
(5) Physical	−0.01 (0.698)
AF-5	−0.12 (<0.001)

The correlation is significant at the *p* ≤ 0.05. Each score obtained on the dimensions is based on a Likert scale (1–5).

## Data Availability

The datasets used during the current study are available from the corresponding author upon reasonable request.
